# Characteristics associated with the satisfaction of users of dental specialty centers

**DOI:** 10.1590/1807-3107bor-2026.vol40.011

**Published:** 2026-05-08

**Authors:** Samuel TREZENA, Allana Ferreira e SILVA, Fabrício Emanuel Soares de OLIVEIRA, Everton Barroso RIOS, Sabina Pena Borges PÊGO, Hercílio MARTELLI, Verônica Oliveira DIAS, Daniella Reis Barbosa MARTELLI

**Affiliations:** (a) Universidade Estadual de Montes Claros - Unimontes, Center of Health and Biological Sciences, Department of Dentistry, Montes Claros, MG, Brazil.; (b) Pontifícia Universidade Católica de Minas Gerais – PUC Minas, Postgraduate Program in Dentistry, Belo Horizonte, MG, Brazil.; (c) Universidade Estadual de Montes Claros - Unimontes, Center of Health and Biological Sciences, Postgraduate Program in Health Sciences, Montes Claros, MG, Brazil.; (d) Universidade Estadual de Montes Claros - Unimontes, Center of Health and Biological Sciences, Postgraduate Program in Primary Care, Montes Claros, MG, Brazil.

**Keywords:** Patient Satisfaction, Dental Health Services, Secondary Care, Quality of Health Care

## Abstract

Dental Specialty Centers (CEOs in Portuguese) are health facilities that offer services of medium complexity in oral health, enabling comprehensive care. The objective of this study is to evaluate the sociodemographic and accessibility characteristics that may be associated with the degree of satisfaction of CEO users. A cross-sectional study was conducted using secondary data from the second cycle of the Program to Improve Access and Quality of CEOs (PMAQ-CEO), available in the public domain on the Ministry of Health website, from 2020. The outcome (satisfaction) was measured by the question “In general, the service you receive in this CEO is?” and Poisson regression analysis was performed using a robust estimator. In total, 10,391 users and 1,042 CEOs were interviewed. More than half of the participants (58.4%) rated the CEO’s service as very good, and 38.2% as “good.” In the final model, greater satisfaction with the CEO was associated (p<0.05) with White individuals who received more than one minimum wage, who did not reside in the same municipality where the establishment operated, who did not want to change the CEO, and with hours of operation compatible with the demands of users. A high percentage of users were satisfied with Brazilian CEOs’ services. By analyzing the literature and data from the previous cycle of the PMAQ-CEO, an improvement in service delivery can be seen, which may have influenced user satisfaction.

## Introduction

The National Oral Health Policy (PNSB - *Política Nacional de Saúde Bucal in Portuguese)*, called Brasil Sorridente, created strategies that enabled the expansion and qualification of the population’s access to actions for the promotion, prevention, recovery, and rehabilitation of oral health, understanding that this is fundamental for the general health and quality of life of the population.^1,2^

These strategies include the implementation of Specialized Care through Dental Specialty Centers (CEOs) and Regional Dental Prosthesis Laboratories (LRPD in Portuguese).^3^ CEOs are a health establishment that offers medium-complexity services in oral health with the objective of enabling comprehensive care, from reference and counter-reference to the Oral Health Teams (eSB in Portuguese) of Primary Health Care (PHC).^1-4^

The implementation of CEOs began in 2004, and as of May 2017, Brazil had 1,078 services in place.^5^ As one of the main objectives of the Ministry of Health is to execute public management based on the induction, monitoring, and evaluation of processes and measurable results, in 2013, the Program to Improve Access and Quality of CEOs (PMAQ-CEO) was established to induce the expansion of access and improve the quality of CEOs.^6^ One of the aims of the program was to guarantee the standardization of these establishments, maintaining a standard of quality at all levels of management and resulting in greater transparency and effectiveness of government actions directed toward specialized oral health care.^5-7^

Thus, it is essential to evaluate the quality of specialized oral health care in Brazil^8,9^ and the level of satisfaction among those who use this service. The results of the first evaluation showed an overall positive assessment of the services (“good” or “very good”), with high satisfaction regarding professionals and the effectiveness of the treatments. However, this initial evaluation also pointed to low familiarity among users with the tools of social control^10^ and identified factors associated with dissatisfaction, such as longer travel and wait times, as well as the importance of welcoming attitudes, clarity in the information provided, and guidance received.^11^ Moreover, an analysis of data from the two cycles of the PMAQ-CEO (2014 and 2016)^12^ showed an increase in the number of CEOs as well as an increase in unmet demand and waiting times, despite improvements in work processes.

A methodological study^13^ aimed at creating a proposal for a user satisfaction index (SSI) identified factors such as shorter waiting times, good conditions for using the CEO, no desire to be treated elsewhere, no interruption of treatment due to lack of materials, age under 40 years, and higher education levels as being associated with higher satisfaction. Additionally, analysis of the association between satisfaction and the structural and process characteristics of CEOs in both cycles of the PMAQ-CEO^14^ revealed that structural characteristics were the most significant in explaining the variance in satisfaction, with better-structured CEOs showing higher satisfaction rates.

Due to the scarcity of studies that have analyzed these variables in the second cycle of the PMAQ-CEO, this study aimed to evaluate the sociodemographic and accessibility characteristics that may be associated with the degree of satisfaction of users with CEOs.

## Methods

### Design

This is a cross-sectional study.

### Data collection

The evaluation carried out by the PMAQ-CEO took place from October to December 2018, in which 85 previously trained evaluators applied instruments to evaluate the CEOs. This external evaluation instrument contained three modules: a) direct observation of the establishment’s infrastructure, b) interviews with the manager and dentist working on site, and c) interviews with the CEOs’ users. Data were collected electronically through tablets and transferred to the Web to consolidate a database available on the Ministry of Health website. This study is based on the answers to Module III (interview with the CEO). The data were downloaded in December 2023.

The interviews aimed to verify users’ perceptions of specialized oral health services regarding the characteristics of access, use, and satisfaction. The questionnaire for Module III was administered to ten users present in each CEO, chosen randomly by the interviewer. The inclusion criteria were users aged 18 years or older who had previously received care from the CEO. Participants were excluded if they were attending their first appointment with the CEO or were present in the unit solely as companions to other patients.

The independent variables selected for this study were related to the sociodemographic profiles of the users and their access characteristics. (1) Sociodemographics, including sex (male/female), age (continuous), self-declared race (White/non-White), marital status (with/without partner), education (≤ 8 years/> 8 years), employment (paid work/unemployed/retired), Bolsa Família participation (yes/no), and family income (BRL, continuous); and (2) Service access, comprising municipality of residence (same/different from CEO), schedule compatibility (yes/no), intent to change CEO (yes/no), residence area (urban/rural), travel time (≤20/>30 min), scheduling method (direct/health unit referral/other), and wait time (≤ 1 week/1 week-1 month/> 1 month). The full variable specifications are detailed in Supplementary Table 1 (questions extracted from the scoring matrix of the PMAQ-CEO External Evaluation (AVE/PMAQ-CEO) used in this study).

**Table 1:** Descriptive statistics of the distribution of user satisfaction with Dental Specialty Centers (CEOs) by federative unit and geographic region, Brazil [n(%)].

Geographic region	Satisfaction with the CEO					Total
	Very good	Good	Regular	Bad	Very bad	n (%)
North						652 (6.3)
Acre	09 (0.1)	09 (0.1)	02 (0.0)	-	-	20 (0.2)
Amazonas	69 (0.7)	58 (0.6)	03 (0.0)	01 (0.0)	-	131 (1.3)
Amapá	08 (0.1)	12 (0.1)	-	-	-	20 (0.2)
Pará	119 (1.1)	175 (1.7)	22 (0.2)	01 (0.0)	02 (0.0)	319 (3.1)
Rondônia	32 (0.3)	41 (0.4)	05 (0.0)	-	-	78 (0.8)
Roraima	10 (0.1)	10 (0.1)	-	-	01 (0.0)	21 (0.2)
Tocantins	16 (0.2)	45 (0.4)	02 (0.0)	-	-	63 (0.6)
Northeast						4.024 (38.7)
Alagoas	125 (1.2)	107 (1.0)	12 (0.1)	-	-	244 (2.3)
Bahia	400 (3.8)	329 (3.2)	42 (0.4)	04 (0.0)	03 (0.0)	778 (7.5)
Ceará	449 (4.3)	253 (2.4)	14 (0.1)	-	01 (0.0)	717 (6.9)
Maranhão	95 (0.9)	166 (1.6)	16 (0.2)	-	-	277 (2.7)
Paraíba	519 (5.0)	274 (2.6)	23 (0.2)	02 (0.0)	-	818 (7.9)
Pernambuco	315 (3.0)	254 (2.4)	12 (0.1)	-	03 (0.0)	584 (5.6)
Piauí	92 (0.9)	155 (1.5)	25 (0.2)	03 (0.0)	04 (0.0)	279 (2.7)
Rio Grande do Norte	119 (1.1)	97 (0.9)	09 (0.1)	01 (0.0)	-	226 (2.2)
Sergipe	39 (0.4)	56 (0.5)	05 (0.0)	-	01 (0.0)	101 (1.0)
Midwest						721 (6.9)
Distrito Federal	56 (0.5)	32 (0.3)	03 (0.0)	-	-	91 (0.9)
Goiás	213 (2.0)	123 (1.2)	05 (0.0)	-	01 (0.0)	342 (3.3)
Mato Grosso do Sul	54 (0.5)	84 (0.8)	10 (0.1)	-	-	148 (1.4)
Mato Grosso	69 (0.7)	66 (0.6)	05 (0.0)	-	-	140 (1.3)
South						1.306 (12.6)
Paraná	258 (2.5)	204 (2.0)	17 (0.2)	-	01 (0.0)	480 (4.6)
Rio Grande do Sul	260 (2.5)	73 (0.7)	07 (0.1)	01 (0.0)	-	341 (3.3)
Santa Catarina	302 (2.9)	177 (1.7)	06 (0.1)	-	-	485 (4.7)
Southeaest						3.688 (35.5)
Espírito Santo	48 (0.5)	40 (0.4)	04 (0.0)	-	-	92 (0.9)
Minas Gerais	630 (6.1)	304 (2.9)	13 (0.1)	-	01 (0.0)	948 (9.1)
Rio de Janeiro	531 (5.1)	204 (2.0)	08 (0.1)	-	02 (0.0)	745 (7.2)
São Paulo	1,234 (11.9)	624 (6.0)	39 (0.4)	04 (0.0)	02 (0.0)	1.903 (18.3)
Total	6,071 (58.4)	3,972 (38.2)	309 (3.0)	18 (0.2)	21 (0.2)	10,391 (100.0)

The outcome investigated (satisfaction with the CEO) was evaluated by the question, “In general, the service you receive in this CEO is?”; the participants chose from “very good,” “good,” “regular,” “bad,” and “very bad.” For the analysis in this study, the answer options were categorized into satisfied (“very good” and “good”) and dissatisfied (“regular,” “bad” and “very bad”).

### Statistical analysis

The data were downloaded in Microsoft Excel^®^ and exported to the Statistical Package for the Social Sciences (SPSS) version 24.0 for statistical analyses.

The first stage consisted of a descriptive analysis of the level of satisfaction with the CEO by state and geographic region. Bivariate analysis and calculation of the crude Prevalence Ratio (PR) with a 95% confidence interval (95%CI) were performed using the chi-squared Pearson test. In the second stage, adjusted PR values were derived using a Poisson regression model with robust variance. The independent variables were dichotomized and/or grouped according to the following description: a) Age: dichotomized using the lower limit of the median as cut-off point; b) Self-declared color race: dichotomized in White and non-White; c) Marital status: dichotomized in with partner and without partner; d) Years of study: dichotomized in up to 08 years of study and more than 08 years; e) Family income: classified taking into account the value of the minimum wage in force in the year of data collection (954.00 BRL) and subsequently dichotomized in up to one minimum wage and more than one minimum wage; f) CEO Access time: dichotomized using the median as a cutoff point; g) CEO access consultation: grouped in “patient agenda directly,” “referencing by Basic Health Unit,” and others; h) appointment/scheduling: grouped into “scheduled time,” “arrival order,” and “others”; and i) scheduling time for attendance at the CEO: grouped in “up to a week,” “from one week to one month,” and “more than a month”.

Multiple analyses were performed using the Poisson regression model with robust variance, and only variables with p-values below 0.20 in the bivariate analysis were included. The model was constructed using the insertion method. Multicollinearity was evaluated using the variance inflation factor (VIF) values, and the goodness of fit of the model was evaluated using the deviance test. Statistical significance was set at p < 0.05. significant.

### Ethical considerations

As anonymous data were used in the public domain, there was no need for assessment by the Research Ethics Committee.

## Results

The interviewees were 10,391 users of the 1,042 CEOs evaluated in the second cycle of the PMAQ-CEO. There was a greater concentration of establishments in the Northeast and Southeast regions, whereas the northern region had the lowest service offer. The state of São Paulo continues to concentrate a greater number of CEOs. Most respondents were satisfied with the services offered by the DSCs; 58.4% rated “very good” and 38.2% as “good.” Less than 1% of the respondents reported that the service was “bad” or “very bad,” with a higher prevalence of this option in the states of Piauí and Bahia (Table 1).

Most respondents were female (67.5%), aged over 30 years (75.2%), lived in urban areas (84.0%), 43.0% had some type of paid activity, 70.6% received more than a minimum wage, and 23.3% were beneficiaries of Bolsa Família. The associations between CEO satisfaction and user characteristics are presented in Table 2. A higher prevalence of CEO satisfaction was perceived by and associated with users over 31 years old, White, with paid work, who received more than one minimum wage, and who did not participate in the Bolsa Família Program.

**Table 2:** Bivariate association between the sociodemographic characteristics of users of Dental Specialty Centers (CEOs) and service satisfaction, Brazil (n = 10,391).

Variable	Dissatisfied	Satisfied		
	N (%)	N (%)	PR* (95%CI)	PR* (95%CI)
Sex				
Male	101 (3.0)	3,275 (97.0)	1.005 (0.99-1.01)	0.160
Female	247 (3.5)	6,768 (96.5)	1	
Age (years)				
18–30	101 (4.1)	2,367 (95.9)	0.99 (0.98-0.99)	0.029**
≥ 31 years	238 (3.2)	7,258 (96.8)	1	
Self-declared color/race				
White	96 (2.6)	3,660 (97.4)	1.01 (1.00-1.02)	0.001**
Non white	252 (3.8)	6,383 (96.2)	1	
Marital status				
With partner	185 (3.4)	5,310 (96.6)	1.01 (0.99-1.007)	0.916
Without partner	163 (3.3)	4,733 (96.7)	1	
Years of study				
< 8	165 (3.7)	4,251 (96.3)	0.99 (0.98-1.00)	0.059
≥ 8	183 (3.1)	5,792 (93.9)	1	
Paid work				
Yes	112 (2.5)	4,363 (97.5)	1.16 (1.01-1.23)	< 0.001**
No	230 (4.0)	5,451 (96.0)	1	
Retired				
Yes	52 (2.9)	1,717 (97.1)	1.05 (0.99-1.01)	0.293
No	296 (3.4)	8,326 (96.6)	1	
Family income				
Up to one minimum wage	85 (4.5)	1,821 (95.5)	0.97 (0.96-0.98)	< 0.001**
Over one minimum wage	110 (2.2)	4,835 (97.8)	1	
Participation in the *Bolsa Família* Program				
No	213 (2.8)	7,513 (97.2)	1.24 (1.14-1.34)	< 0.001**
Yes	127 (5.1)	2,380 (94.9)	1	
Followed-up by the Family Health Strategy				
Yes	270 (3.2)	8,047 (96.8)	1.04 (0.99-1.14)	0.355
No	67 (3.7)	1,756 (96.3)	1	

*Crude Prevalence Ratio;**Statistical significance with Pearson’s chi-square test.

Most respondents lived in the same municipality as the CEO (92.4%), reported that the establishment operated at a time compatible with their demands (96.2%), and took less than one month to schedule their appointments (68.1%). The most prevalent scheduling method was directly established by the user (65.3%), and most respondents (88.5%) stated that they would not change their CEOs if given the opportunity. Among the 1,196 respondents who expressed a desire to change CEOs, the primary reason cited was distance (57.9%), followed by the conditions of the establishment’s infrastructure and the lack of professionals (21.2%). Other reasons accounted for 11.3% of the responses; 4.5% reported poor treatment, and 2.5% cited inconvenient service hours. These findings highlight that most users find the location, scheduling, and operating hours of CEOs convenient. However, for those who expressed dissatisfaction, distance and infrastructure issues were the primary barriers.

Regarding variables related to access, statistically significant associations were observed for almost all factors, except for area of residence. A higher satisfaction prevalence was identified among users who accessed the CEO within 20 minutes (20%), scheduled their appointment directly (20%), and received consultation-based services (23%). Table 3 presents the results of the bivariate analysis of user satisfaction and service access characteristics. The strong association between shorter travel times, direct scheduling, consultation-based services, and higher satisfaction underscores the importance of convenience and user autonomy in shaping positive experiences.

**Table 3:** Bivariate association between user satisfaction of Dental Specialty Centers (CEOs) and characteristics of access to services, Brazil.

Variable	Dissatisfied	Satisfied	PR* (95%CI)	p-value
	n (%)	n (%)		
Reside in the municipality where the CEO is located				
Yes	333 (3.5)	9,269 (96.5)	0.98 (0.97-0.99)	0.019**
No	15 (1.9)	774 (98.1)	1	
Area of residence				
Urban	275 (3.3)	8,401 (96.7)	1.08 (0.99-1.019)	0.107
Rural	65 (3.9)	1,584 (96.1)	1	
CEO opening hours compatible with your demands				
Yes	288 (2.9)	9,705 (97.1)	1.14 (1.09-1.19)	< 0.001**
No	60 (15.1)	338 (84.9)	1	
Would like to change the CEO				
Yes	174 (14.5)	1,022 (85.5)	0.87 (0.85-0.89)	< 0.001**
No	174 (1.9)	9,021 (98.1)	1	
Time to access the CEO				
Up to 20 minutes	171 (2.7)	6,217 (97.3)	1.20 (1.12-1.29)	< 0.001**
Over half na hour	171 (4.6)	3,538 (95.4)	1	
Access consultation to the CEO				
Patient schedules directly	271 (4.0)	6,488 (96.0)	1.20 (1.13-1.27)	< 0.001**
Referral by the BHU	74 (2.3)	3,249 (97.7)	1.01 (1.00-1.02)	0.137
Other	02 (0.8)	256 (99.2)	1	
Consultation booking/Service				
Appointment time	111 (2.3)	4,903 (97.7)	1.023 (1.016-1.030)	< 0.001**
Order of arrival	64 (4.3)	1,427 (95.7)	0.98 (0.97-0.99)	0.027**
Others	171 (4.5)	3,687 (95.5)	1	
Appointment time for appointment at CEO				
Up to one week	79 (2.5)	3,119 (97.5)	1.13 (1.00-1.20)	< 0.001**
From one week to one month	97 (2.6)	3,616 (97.4)	1.12 (1.05-1.19)	0.002**
Over a month	161 (5.0)	3,071 (95.0)	1	

*Crude prevalence ratio; **Statistical significance with Pearson’s chi-square test.

Table 4 presents the adjusted model composed of variables that maintained statistical significance in multiple analyses. It is perceived that among the sociodemographic characteristics, only White (PR: 1.011, 95%CI: 1.000–1.018) and income above a minimum wage (PR: 0.983, 95%CI: 0.973–0.993) remained associated. Additionally, of the seven variables that presented significance in the bivariate analysis, only three constituted the final model: not residing in the same municipality as the CEO (PR:1.824 95%CI: 1.093–3.044); hours of operation compatible with the demands of the user (PR: 1.090, 95%CI: 1.042–1.142), and would not change the CEO (PR: 7.688, 95%CI: 6.286–9.402).

**Table 4:** Multiple analysis by Poisson regression with robust variance model between sociodemographic characteristics and access to services of users, with satisfaction with Dental Specialty Centers (CEOs), Brazil.

Variable	PR**	95%CI	p-value**
Self-declared color/race			
White	1.011	1.00-1.018	0.006***
Non white	1		
Family income			
Up to one minimum wage	0.983	0.973-0.993	0.001***
Over one minimum wage	1		
Reside in the municipality where the CEO is located			
Yes	0.980	0.968-0.991	< 0.001***
No	1		
CEO opening hours compatible with your demands			
Yes	1.090	1.042-1.142	< 0.001***
No	1		
Would like to change the CEO			
Yes	0.895	0.872-0.918	< 0.001***
No	1		

*Adjusted prevalence ratio; **Poisson regression with robust variance; ***Statistical significance;

## Discussion

The results revealed that most users were satisfied with the services provided by CEOs, offering a comprehensive perspective on geographical distribution and associated factors. Poisson regression analysis identified the key factors associated with higher satisfaction, including being White, having a monthly family income above one minimum wage, not residing in the same municipality as the CEO, having operating hours compatible with user demands, and expressing no desire to change the CEO. These findings highlight the importance of socioeconomic factors, accessibility, and user-centered service organizations in shaping satisfaction levels. Additionally, the strong association between direct scheduling, shorter travel times, and consultation-based services with higher satisfaction underscores the role of convenience and autonomy in enhancing user experience.

Notably, less than 1% of the users rated the service as “bad” or “very bad,” reflecting a significant reduction in dissatisfaction rates from 4.8% in the first cycle to 0.4% in the second cycle. This decline aligns with the findings of Andrade and Pinto,^11^ who identified factors such as long waiting times and inadequate infrastructure as the key drivers of user dissatisfaction. Furthermore, the proportion of users who rated the service as “very good” increased by just over 10%, a trend consistent with the findings of Cavalcanti, Cardoso and Padilha.^10^ Their analysis of the PMAQ-CEO data highlighted that user satisfaction was closely tied to the resolution of treatment and the quality of care provided by dental professionals. These findings underscore the importance of maintaining high standards of clinical care and ensuring that patients perceive treatment as effective. Recent studies have used the satisfaction data of the second cycle of the PMAQ-CEO to create a satisfaction index^13^and associated it with the structure and work processes of the establishments,^14^ identifying the maximum grade assignment with the shortest waiting time for treatment start, good conditions of use, willingness not to change the CEO,^14^ users assisted by units that organized their demands from primary care referrals, and those that often participated in continuing education actions.^13,15^

The highest prevalence of dissatisfaction was observed in the states of Piauí and Bahia, indicating possible areas for localized improvement. No studies have associated user satisfaction with Brazilian federal units; however, it is interesting to note that the significant concentration of CEOs in the Northeast and Southeast regions, with a notably lower number in the North region, is perceived as a challenge to accessibility that can directly affect populations with limited assistance.^12,16,17^

These regional disparities are further compounded by socioeconomic inequalities, as municipalities with better social indicators, such as higher human development indices (HDI) and schooling rates, tend to demonstrate better service performance and higher user satisfaction.^18^ This finding suggests that improving access to CEOs in regions with lower HDI and educational levels could help reduce inequalities and enhance satisfaction among users.^18^ Expanding the availability of CEOs, particularly in underserved areas such as the North and Central-West regions, could alleviate repressed demand, improve service resolvability, and ultimately lead to greater user satisfaction^19^. Public policies should prioritize investments in infrastructure and workforce distribution to address these disparities and ensure equitable access to specialized dental care across all regions.^10,12,18,19^

Studies have associated higher ages and satisfaction with CEO,^13,14,20^ with a similar result to the bivariate analysis performed in this study. Among the variables characterizing users, only being White and monthly income greater than the minimum wage remained in the final regression model. Previous studies^21^ have identified higher levels of dissatisfaction with oral health services in non-White individuals who received less than 1,500.00 BRL monthly, which may be a result of social factors related to the marginalization of the Black population, with fewer opportunities for work and access to education, and consequent difficulty in accessing oral health services. Racial issues were also associated with the satisfaction of users of oral health services in Brazilian primary care, with the White population being more satisfied than the self-declared Brown, Black, and Yellow.^20^

These findings align with recent research^22^ that evaluated the role of active health ombudsman services for CEOs, demonstrating that such mechanisms can reduce racial inequalities in access, reception, and user satisfaction. Specifically, CEOs that implemented active ombudsman services for planning and user feedback showed lower racial disparities in service quality and user experience.^22^ This suggests that expanding the use of ombudsman services by CEOs could be an effective strategy to address racial and socioeconomic barriers and ensure more equitable access to specialized dental care. Therefore, targeted policies are required to strengthen these mechanisms, improve infrastructure, and implement inclusive practices that prioritize the needs of historically underserved populations.

Regarding the analyses related to the skin color of users, the binary racial categorization between “White” and “non-White” may be a limitation of the study, as it may not fully capture the country’s racial and ethnic diversity. However, it is important to clarify that this categorization is widely used in national and international research to correlate factors that can be attributed to users of health services in relation to “white privilege.”

The profile associated with satisfaction with CEO services showed a higher prevalence of White users, with paid work, higher family income, and nonparticipation in the Bolsa Família Program. The behavior of these variables may have suffered from demographic and socioeconomic influences, mainly because they are public services. Other points that can be considered are the greater financial access of these individuals to establishments, greater awareness of the importance of oral health, and aspects of cultural barriers and social exclusion.^16,17,20,21^

These findings are consistent with the study by Cunha et al.,^23^ which identified that beneficiaries of the Bolsa Família program and individuals without coverage by the Family Health Strategy (FHS) were more likely to miss scheduled appointments, highlighting the challenges faced by vulnerable populations in accessing specialized dental care. Poor facility conditions and a lack of integration with FHS were also associated with higher absenteeism, suggesting that improving infrastructure and strengthening coordination between primary and specialized care could reduce barriers to access and enhance user satisfaction.

Moreover, the integration of Primary Health Care (PHC) and CEOs has been shown to play a critical role in improving user satisfaction. Silva et al.^15^ demonstrated that the regulation of CEO access by PHC improved service organization, reduced waiting times, and enhanced user satisfaction. By acting as a filter, PHC can ensure that users reach CEOs with clear treatment needs, improving service resolvability and reducing unnecessary demand. This underscores the importance of strengthening coordination between primary and specialized care to optimize service delivery and user experience.

Interestingly, not residing in the same municipality as the CEO showed an association with service satisfaction, contrary to studies that associate shorter distances of access and satisfaction with oral health services.^24-27^ Cunha et al.^28^observed that individuals treated for the manufacture of total prostheses, in the vast majority, resided in the same city as the establishment and positively rated the embracement and service at the CEO, which can be attributed to the shortest distance for care. Contrarily, the study by Costa, Carneiro and Oliveria^29^ conducted with the CEO in Ceará, inferred that the distance of the CEO did not interfere with user satisfaction, similar to the findings of this study. Nevertheless, this variable should be analyzed with caution, since the time of access to the CEO below 20 minutes presented a 20% higher prevalence associated with user satisfaction with the CEO than the time of access of more than half an hour. There is no apparent justification for the contrary behavior of these variables; however, contextual and subjective factors, such as ease of physical access to the place, influence of the service provided by the establishments, nearby municipalities, and even transport financed by public management, may influence the perception of satisfaction in relation to these two variables.

Better satisfaction was observed among users who stated that CEOs’ operating hours were compatible with their demands. The increase in the number of specialists and their hourly loads from the first to the second cycle of the PMAQ-CEO^12,30^ may have been one of the factors that contributed to the compatibility of the operation of establishments with the demands of users, and consequently affected satisfaction. Establishments with greater provision and flexibility of schedules facilitate access to the service, shorter waiting times, punctuality in appearance, and reduction in scheduling time.

In the first cycle of PMAQ-CEO, the North and Midwest regions presented the lowest absolute number of establishments, with an average travel time of 28.4 minutes, and the intention to change the CEO due to distance from home was reported by 7.8% of users^17^. The second-cycle data described in this study showed that the willingness to change CEO due to distance was 6.7%, with a reduction of just over 15% of the responses. This change may have been influenced by the increase in the number of accredited establishments after the first cycle of evaluation,^12,16^ as well as by the improvement of services provided, among other factors that also corroborated higher satisfaction with the services. In addition, the desire not to be met by another CEO had an odds ratio of 4.17 (95% CI: 3.12-5.57), with higher satisfaction rates.^13^

Even though they did not remain in the final model, the variables “access consultation,” “consultation/service booking,” and “scheduling time for service at the CEO” showed statistical associations with user satisfaction. Notably, a relationship exists between these variables. Allowing customers to schedule their service at the time of their choice implies shorter waiting times and greater organization of the establishment, thus influencing the satisfactory experience and perception of the user with the service. However, this is not a reality on a national scale.

A study^12^ analyzing both cycles of the PMAQ-CEO found a significant increase in the demand for service at the CEO, even with an increase in units and professionals. Analyses regarding the waiting time for scheduling procedures and specialized consultations in the CEO^31,32^ found that local factors related to labor processes and human resources, such as finances and infrastructure, are determinants of greater agility in scheduling. Therefore, it is necessary to investigate the impact of the organization of the work processes of CEOs on the efficiency of the work provided, since access to the service is a primary factor in user experience.

One limitation of the present study is the potential selection bias, given that the participants were approached at the CEO site on the day of their consultation. This setting may have influenced their responses, as individuals may have felt compelled to report either positive or negative feedback. On the one hand, users might hesitate to express dissatisfaction because of concerns about affecting their current or future care. On the other hand, they might also feel pressured to provide positive responses out of gratitude or a desire to please healthcare providers. This dual possibility highlights the complexity of collecting feedback in healthcare settings and underscores the need for future studies to employ more neutral or anonymous methods of data collection to minimize such biases. Moreover, less-satisfied individuals are more predisposed to abandon their treatment.^11^ Despite these limitations, this study provides valuable insights into user satisfaction and areas requiring improvement in the delivery of specialized oral health services at a national level. Furthermore, it is interesting to note that the services offered in quantitative terms were not directly related to improved access for the population.

Although this study provides valuable insights into user satisfaction with CEOs in Brazil, future research could benefit from adopting complementary methodologies to deepen the understanding of these findings. Longitudinal studies can track changes in user satisfaction over time, particularly in response to policy changes, infrastructure improvements, or shifts in service delivery models. Such studies would help identify whether the factors associated with satisfaction remain consistent or evolve as healthcare systems adapt. Additionally, qualitative research can explore the subjective experiences of users, shedding light on the contextual and cultural factors that influence satisfaction beyond what quantitative metrics can capture. Combining these approaches will provide a more comprehensive understanding of user satisfaction and inform targeted interventions to improve the quality and equity of CEO services.

## Conclusion

This study identifies a high prevalence of user satisfaction with the services offered by CEOs in Brazil. The key factors associated with higher satisfaction included being White, having a monthly family income above the minimum wage, residing outside the municipality in which the CEO was located, having schedules compatible with user demands, and expressing no desire to change CEOs. These findings highlight the importance of addressing socioeconomic and logistical factors to enhance user experience and ensure equitable access to dental care services.

The practical implications of these results are significant for improving CEO services. For instance, optimizing scheduling processes to better align with user availability and addressing barriers related to income and geographic location could further enhance satisfaction. Additionally, efforts to reduce disparities in access to and quality of care for non-White and lower-income groups are essential for promoting equity in dental health services. By focusing on these factors, policymakers and healthcare managers can better organize local work processes, resolve unmet demands, and facilitate broader access to specialized dental care.

## Data Availability

The content will be available when the article is published.
